# Adjustment of the velocity encoding parameter to the blood flow velocity is not necessary for accurate and precise quantification of aortic regurgitation severity with phase contrast magnetic resonance imaging

**DOI:** 10.1186/1532-429X-17-S1-P329

**Published:** 2015-02-03

**Authors:** Frida Svensson, Christian L Polte, Åse A Johnsson, Sinsia A Gao, Odd Bech-Hanssen, Kerstin Lagerstrand

**Affiliations:** 1 Medical Physics and Technology, Sahlgrenska University Hospital, Gothenburg, Sweden; 2 Clinical Physiology, Sahlgrenska University Hospital, Gothenburg, Sweden; 3 Cardiology, Sahlgrenska University Hospital, Gothenburg, Sweden; 4 Radiology, Sahlgrenska University Hospital, Gothenburg, Sweden

## Background

Accurate and precise quantification of aortic regurgitation (AVR) severity by cardiovascular magnetic resonance is essential for the clinical decission-making and timing of surgery. The regurgitant flow volume (RV) can be measured directly by 2D phase contrast (PC) velocity measurements. The velocity encoding parameter, venc, has been identified by others as an important factor for accurate and precise determination of RV. For large vessel and high signal to noise measurements, though, integration of the measured blood flow velocities over the vessel lumen and over the cardiac phases for calculation of RV should average out variations in the measured velocities and enable high precision estimates of RV independently of venc. Furthermore, application of a correction method that effectively reduces the background velocity offset in the PC image to a sufficiently low value should enable accurate estimation of RV and remove the venc dependency. The aim of the study was to demonstrate venc insensitivity in the estimated RV with effectively background offset corrected PC velocity measurements.

## Methods

Measurements with high and low venc (~150 and 50 cm/s, respectively) were performed at the sinotubular junction on patients (n=28; 27-83y) and volunteers (n=26; 24-58y) using a 1.5 T scanner. Corrections for background offsets were automatically performed by the scanner and post-acquisition by means of adaptive image filtering. The mean background offset, standard deviation (sd) and coefficient of variation (cv) of repeated measurements were determined for the whole cohort. The mean RV, and sd and cv of RV were determined for patients and volunteers separately. For comparison, Wilcoxon signed-rank test was performed at a significance level of p<0.05.

## Results

**Figure 1:**
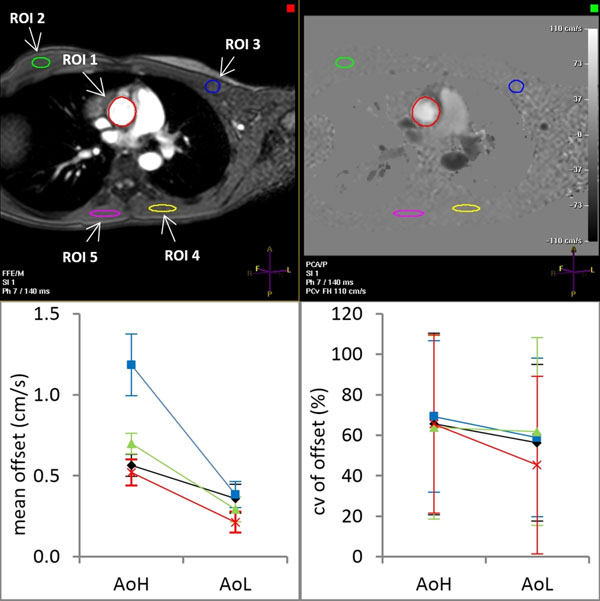
**The background velocity offset after correction determined in ROI2 (◊), ROI3 (□), ROI4 (Δ) and ROI5 (×) in stationary muscle tissue for the high (AoH) and low venc measurement (AoL).** Error bars in the mean show the error propagated sd from repeated measurements of individual subjects and error bars in cv show the sd among all subjects.

**Figure 2:**
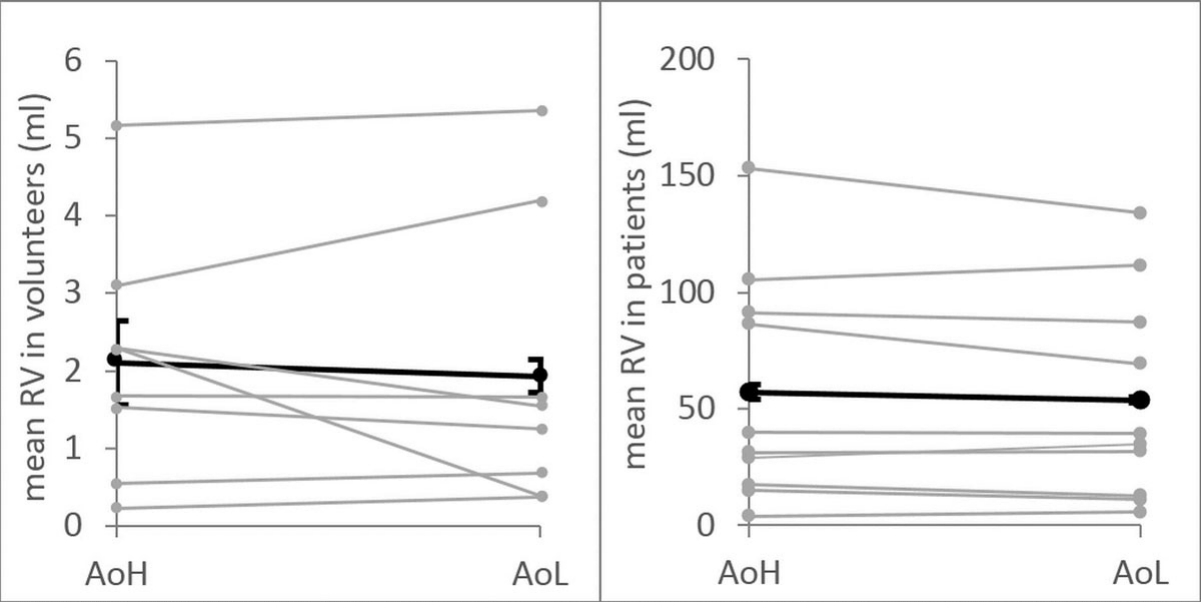
**The RV in volunteers (right) and patients (left) after correction for the high (AoH) and low venc measurement (AoL).** Grey dots show the mean of repeated measurements of each subject and black dots show the mean of all subjects. Error bars, shown as the sd of the mean propagated from repeated measurements in individual subjects, was at some points smaller than the symbols.

A background offset was present in all measurements. After correction, the offset was small (Fig 1) and did not cause any significant differences in RV regarding venc (p>0.8; Fig 2). Also, the variation in RV, determined from repeated measurements, did not significantly depend on venc (p>0.8).

## Conclusions

We have demonstrated venc insensitivity in both the accuracy and precision of RV using background offset corrected PC velocity measurements. Without the need for adjustment of venc to the blood flow velocity, the time for the examination will be substantially reduced.

## Funding

This study was funded by a project grant from the Health & Medical Care Committee of the Regional Executive Board, Västra Götaland Region, Sweden.

